# Polyelectrolyte Multilayer Films as a Potential Buccal Platform for Drug Delivery

**DOI:** 10.3390/polym14040734

**Published:** 2022-02-14

**Authors:** Bissera Pilicheva, Yordanka Uzunova, Maria Marudova

**Affiliations:** 1 Department of Pharmaceutical Sciences, Faculty of Pharmacy, Medical University of Plovdiv, 15A Vassil Aprilov Blvd, 4002 Plovdiv, Bulgaria; 2 Research Institute, Medical University of Plovdiv, 15A Vassil Aprilov Blvd, 4002 Plovdiv, Bulgaria; yordanka.uzunova@mu-plovdiv.bg; 3 Department of Bioorganic Chemistry, Faculty of Pharmacy, Medical University of Plovdiv, 15A Vassil Aprilov Blvd, 4002 Plovdiv, Bulgaria; 4 Department of Physics, Faculty of Physics and Technology, University of Plovdiv “Paisii Hilendarski”, 24 Tzar Asen Str., 4000 Plovdiv, Bulgaria; marudova@uni-plovdiv.net

**Keywords:** multilayer films, polyelectrolytes, buccal drug delivery, benzydamine, tolfenamic acid, betahistine, mucoadhesion

## Abstract

The goal of this research was to study the potential of polyelectrolyte multilayers as buccal dosage forms for drug delivery and to investigate how the properties of the drugs impact the overall performance of the delivery system. Multilayer films based on the polyelectrolyte interaction between casein and chitosan were developed using benzydamine, tolfenamic acid and betahistine as model drugs. The samples were characterized for surface pH, moisture content and moisture absorption, swelling behavior and mucoadhesion. Additionally, surface morphology was investigated, as well as the drugs’ physical state after incorporation in the multilayer films. The samples proved to be non-irritant (pH was within the physiological range), physically stable (moisture content and moisture absorption below 5%) and mucoadhesive, adsorbing from 60 to 70% mucin. The release behavior corelated to the swelling index profiles of the samples and was strongly dependent on the drug solubility. The developed multilayer films appeared to be an optimum delivery system for sparingly soluble drugs due to the high drug loading achieved.

## 1. Introduction

Contemporary strategies to deliver systemically acting drugs include administration via alternative delivery routes. Several mucosal surfaces have been investigated as delivery routes including nasal, rectal, vaginal, ocular, and oral. The buccal route of administration has been extensively studied for systemic drug delivery due to advantages such as direct access to systemic circulation and bypassing the hepatic first pass effect [1,2]. Additionally, buccal mucosa is easily accessible and relatively immobile, allowing prolonged contact with the dosage form. Moreover, buccal delivery has a high patient compliance [3,4].

The development of buccal dosage forms has increased dramatically over the recent years. Liquid solutions or suspensions, effervescent buccal discs, tablets, and lozenges have been designed with optimized characteristics for buccal application [5,6,7]. Buccal films have gained relevance as novel, convenient and patient-friendly formulations [8]. These formulations have been developed to release drugs locally for the treatment of fungal or bacterial infections [9,10,11,12]. Mucoadhesive films, in turn, provide intimate contact with buccal mucosa, which implies longer retention time and can therefore release drugs in a prolonged manner [13,14]. Furthermore, they improve patient compliance due to their small size, reduced thickness, and refined mechanical properties [15]. Depending on the manufacturing method, drug release from such dosage forms can be oriented either towards the oral cavity (local effect) or towards the buccal mucosa, allowing systemic absorption. Typically, films intended for the systemic delivery of drugs consist of an impermeable backing layer, a drug-containing reservoir, and a mucoadhesive layer for attachment to the mucosa [16]. A common formulation challenge is to increase drug loading into the reservoir layer and to achieve controlled drug release through this layer.

Multilayer films, which are usually prepared using a self-assembly technique, are an emerging field of drug delivery and a promising option to achieve the desired goals [9]. Typically, multilayer films are based on polyelectrolyte linkage between oppositely charged polymer molecules. Drug molecules are usually incorporated during the self-assembly process providing a uniform loading in the multilayer structure. The multilayers, on the other hand, due to the versatile permeability and porosity of the polymers, can hinder the drug diffusion leading to prolonged drug release [17].

Recently, polyelectrolyte complexes of polysaccharides and proteins have been extensively investigated for biomedical applications [18,19]. Chitosan-based complexes have proved to be favorable in various aspects. In acidic medium, because of protonation of the amino groups on the polymer backbone, chitosan becomes a cationic polyelectrolyte capable of interacting with negatively charged polyelectrolytes [20]. Casein, being a natural protein and an anionic polyelectrolyte, is considered a suitable candidate for the development of polyelectrolyte complexes as drug delivery systems [21].

The goal of this research was to study the potential of polyelectrolyte multilayers as buccal dosage forms for drug delivery. In multiple previous studies of our research group, we have thoroughly investigated the influence of the process variables (pH, ionic strength, number of layers, type of the polymers) on the loading and release of benzydamine dihydrochloride, a locally acting drug. Moreover, we evaluated the effect of the polymer crosslinking mode on the drug loading and release. As a result of our studies, we have derived optimal formulation conditions and have outlined a promising model for the investigation of the biopharmaceutical behavior of systemically acting drugs. In the current research, we used the optimized model of polyelectrolyte multilayer films as a delivery system on one hand, and benzydamine hydrochloride (BZ), tolfenamic acid (TA), and betahistine dihydrochloride (BET) as model drugs. TA and BET are systemically acting drugs and are suitable for alternative routes of administration but differ significantly in physical and chemical properties. The aim of this study was to investigate whether the properties of the drugs are determinative for the overall performance of the delivery system, and whether the developed multilayer films can serve as a universal delivery platform for buccal administration.

Benzydamine hydrochloride (BZ) is an indazole non-steroidal anti-inflammatory drug (NSAID) with analgesic, antipyretic, and anti-edema properties. Unlike other NSAIDs, BZ does not inhibit cyclooxygenases but stabilizes membranes, resulting in local anesthesia. Moreover, it exhibits antimicrobial properties. BZ is a solid crystalline low molecular weight hydrophilic drug (Mm 309.4 g/mol), freely soluble in water, alcohol, methanol, and other organic solvents [22].

Tolfenamic acid (TA) is a NSAID with anti-inflammatory, antipyretic, analgesic, and potential anti-neoplastic activities. It is a crystalline, low molecular weight lipophilic drug (Mm 261.70 g/mol) that is slightly soluble in water but freely soluble in buffers with pH > 8 and in organic solvents such as ethanol, DMSO, methanol, acetone, and acetonitrile [23]. TA pharmacokinetics is marked by delayed oral absorption and extensive first-pass metabolism, which make it a suitable drug for an alternative route of administration [24].

Betahistine dihydrochloride (BET) is a histamine analogue used for the symptomatic treatment of vestibular disorders of central and peripheral origin. BET shows a plasma half-life of 3.4 h, which requires frequent dosing and may lead to non-compliance, especially in elderly patients. BET is also a low-molecular weight drug (Mm 209.11 g/mol), but as opposed to TA it is freely soluble in water and ethanol [25]. It was thus challenging to design buccal formulations of TA and BET via optimized technological approach and investigate the impact of the drug properties on the vehicle biopharmaceutical performance.

## 2. Materials and Methods

Poly (DL-lactic acid) with inherent viscosity 0.55–0.75 dL/g was supplied from Lactel Absorbable Polymers (Birmingham, AL, USA). Chitosan (low molar mass, degree of deacetylation > 75%), casein sodium salt from bovine milk, benzydamine hydrochloride, betahistine dihydrochloride, mucin (type II, from porcine stomach) and Bradford reagent were purchased from Sigma-Aldrich GmbH (Taufkirchen, Germany). Tolfenamic acid was delivered by Cayman Chemical Europe (Tallinn, Estonia). All other chemicals were of analytical grade.

### 2.1. Preparation of Poly (DL-Lactide) (PDLA) Backing Layers

PDLA backing layers (approximate thickness 40 μm) were prepared by casting method from 2% *w*/*v* chloroform solution. The films were then dried at room temperature until constant weight and were kept in desiccator at room temperature and relative humidity (RH) of 54%. Afterwards, the substrates were charged in positive corona by means of a point-to-plain three-electrode corona discharge system (+5 kV corona electrode voltage and +1kV grid voltage, for 1 min, at 21–23 °C and RH 40–60%). The prepared PDLA layers were further used as supports for the casein/chitosan multilayers deposition.

### 2.2. Deposition of Polyelectrolyte Multilayers

The multilayers were built up using layer-by-layer (LbL) deposition technique with programmable slide stainer (Poly Stainer IUL, Barcelona, Spain) under the following deposition program: 15 min adsorption of polyelectrolyte solution followed by 5 min washing in distilled water. For the LbL assembly 1% *w*/*v* casein solution in phosphate buffer (pH = 8, ionic strength 100 mM), and 1% *w*/*v* chitosan solution in acetate buffer (pH = 4, ionic strength 100 mM) were prepared. For the deposition, the corona pre-treated PDLA substrates were alternatively dip-coated into the polymers’ solutions. Casein solution was deposited first because of its negative charge so that it can interact with the positively charged support. Each polyelectrolyte deposition step was followed by rinsing in distilled water. The procedure was repeated until eight casein/chitosan layers were obtained. Chitosan was deposited as the final layer due to its ability to attach to mucosal surfaces. For drug loading, the model drug TA was dissolved at a concentration of 1% *w*/*v* into the casein solution (pH = 8), whereas BET and BZ were dissolved in the chitosan solution (pH = 4) at the same concentration. After the deposition of the last layer the multilayer films were dried in hot air and were stored for further analyses in a desiccator at room temperature and 55% RH.

### 2.3. Characterisation of the Multilayer Films

#### 2.3.1. Surface pH

The surface pH of the developed multilayer films was examined to investigate the likelihood of buccal mucosal irritation due to acidic or alkaline pH. A method adapted from Bottenberg et al. was used to determine the surface pH of the films [26]. The samples (2.50 cm × 1.25 cm) were allowed to swell by keeping them in contact with 2 mL of distilled water (pH = 6.5 ± 0.05) for one hour at room temperature. The measurements were performed after bringing the electrode of a pH meter (WTW InoLab 720, Prague, Czech Republic) in contact with the microenvironment of the swollen films and allowing it to equilibrate for one minute. The average pH of three determinations was reported.

#### 2.3.2. Moisture Content

The moisture content was investigated to check the integrity of films in dry conditions. The films (2.50 cm × 1.25 cm) were weighed individually and kept in a desiccator containing anhydrous calcium chloride at room temperature. After 72 h the films were weighed again. The average percentage moisture content of three films was calculated using the following formula:

(1) $$Moisture\;content\;\left(\%\right)=\frac{Initial\;weight-Final\;weight}{Final\;weight}*100$$

#### 2.3.3. Moisture Absorption

The percentage moisture absorption was studied to test the physical stability of the films at extremely high humidity environment. The samples (2.50 cm × 1.25 cm) were weighed accurately and placed in a desiccator where they were exposed to 84% RH using a saturated solution of potassium chloride. The samples were left to equilibrate for 72 h before new weight measurements with an analytical balance were performed. The average percentage moisture absorption of three samples was calculated according to the following equation:

(2) $$Moisture\;absorption\;\left(\%\right)=\frac{Final\;weight-Initial\;weight}{Initial\;weight}*100$$

#### 2.3.4. Swelling Index Determination

The swelling behavior of the multilayer films was investigated in simulated saliva solution (PBS, pH = 6.75). The samples (1 cm × 1 cm) were weighed on an analytical balance (*Wo*) and soaked into a Petri dish containing 5 mL of the medium. At specific time intervals, the films were taken out, the excessive medium was removed carefully with filter paper and the swollen films were weighed again (*Wt*). Swelling Index percentage was calculated using the equation:

(3) $$Swelling\;Index\;\left(\%\right)=\frac{Wt-Wo}{Wo}*100$$

where *Wt* is the weight of the PEM at time *t*, and *Wo* is the initial film weight. The experiments were performed in triplicate.

#### 2.3.5. In Vitro Mucoadhesion Study

Mucoadhesive properties of the multilayers were analyzed based on their mucin binding capacity. The samples (1 cm × 2 cm) were soaked in 2 mL mucin aqueous solution (0.5 mg/mL) for 2 h. Thereafter, the samples were removed, and the amount of unbound mucin was determined using a Bradford colorimetric method [27]. The mucin content was calculated from a standard calibration curve. The percentage of mucin adsorbed on the films’ surface was calculated from the difference between the initial amount of mucin and the amount of free mucin in the medium. The study was performed in triplicates and the mean ± SD values were calculated.

#### 2.3.6. Mechanical Properties

The mechanical properties of the multilayer films were studied at uniaxial tensile deformation at a constant speed of 0.1 mm/s using an LS 1 dynamometer (Lloyd Instruments, Bognor Regis, UK). Stress at break, strain at break and Young’s modulus were calculated from the stress-strain curve.

#### 2.3.7. Fourier Transformed Infrared Spectroscopy (FTIR)

The multilayer structures were evaluated for drug/polymer interactions by Fourier-transformed infrared spectroscopy. The spectra were collected using a Nicolet iS 10 FTIR spectrometer (Thermo Fisher Scientific, Pittsburgh, PA, USA), equipped with a diamond attenuated total reflection (ATR) accessory, operating in the range from 650 cm^−1^ to 4000 cm^−1^ with a resolution of 4 cm^−1^ and 64 scans.

#### 2.3.8. Atomic Force Microscopy (AFM)

The surface morphology of the polyelectrolyte multilayer films was investigated by atomic force microscopy in tapping mode. The experiments were done by Nanosurf flex AFM (Nanosurf, Liestal, Switzerland), equipped with standard cantilevers Tap190Al-G The tip radius was 10 nm. The area of the images was 10 × 10 µm^2^. The mean square root roughness, *Rq* of the surface was calculated based on the dependence [28]:

(4) $$R_{q}=\sqrt{\frac{1}{n}\sum_{i=1}^{n}Z_{i}{}^{2}}$$

where *n* is the total number of data points and *Z_i_* is the height of a data point above the average picture level.

#### 2.3.9. Scanning Electron Microscopy (SEM)

The morphology of the obtained polyelectrolyte multilayer structures was examined by means of SEM. A scanning electron microscope Lyra 3XMU (Tescan, Brno, Czech Republic) was employed. The working voltage was 30 kV. Prior to the measurements, the samples were covered with a thin film of gold (about 30 nm).

#### 2.3.10. Differential Scanning Calorimetry (DSC)

The drug phase state was examined by the method of differential scanning calorimetry. It was performed on a DSC 204F1 Phoenix (Netzsch Gerätebau GmbH, Selb, Germany) at a heating rate of 10 °C/min for a temperature ranging from 25 to 250 °C in an argon environment with a purging rate of 20 mL/min. Aluminum containers were used to seal the samples. The onset temperature, peak temperature, and normalized enthalpy (calibrated against Indium melting enthalpy standard) were analyzed by Netzsch Proteus–Thermal Analysis software 6.1.0B.

#### 2.3.11. Determination of Drug Content in the Multilayer Films

The drug-loaded films (2 cm × 2 cm) were cut into small pieces and were placed into 20 mL phosphate buffer saline (pH = 6.8). Then the samples were stirred on a magnetic stirrer, sonicated for 5 min, and filtered using Chromafil^®^ syringe filter (0.45 μm). The amount of model drugs was determined using HPLC chromatographic system Varian ProStar 325 (Palo Alto, CA, USA) with PDA 335 detector, and Hypersil GOLD C18 column (150 × 4.6 mm, 5 μm) (Thermo Fisher Scientific, Pittsburgh, PA, USA) under the conditions presented in Table 1. The pump supplied a 1 mL/min flow rate of the mobile phase. The chromatograms were recorded for 6 min at 25 °C, and the retention time is shown in Table 1. The drug concentration was calculated from a standard calibration curve of the drugs in mobile phase using Star Chromatography Workstation software (version 6.30). Mean results of triplicate measurements and standard deviation were reported.

#### 2.3.12. In Vitro Drug Release

The dissolution study was carried out using the stirred beaker method. Samples (2 cm × 2 cm, *n* = 3) were placed into a beaker containing 20 mL dissolution media (phosphate buffer saline, pH = 6.8). The experiment was performed at 37 ± 0.5 °C, with a rotation speed of 50 rpm. At predetermined time intervals samples were withdrawn and replaced with an equivalent volume of fresh medium. The withdrawn samples were filtered and analyzed for drug content by HPLC as mentioned above. The mean results of triplicate measurements and standard deviation were reported.

## 3. Results and Discussion

In the present study, multilayer films based on the polyelectrolyte interaction between casein and chitosan were developed. The multilayers were deposited onto a PDLA supports which were pre-treated on a positive corona to allow attachment of the first polyelectrolyte layer, casein. Benzydamine hydrochloride, tolfenamic acid and betahistine dihydrochloride were incorporated in the polymer structures according to their solubility and their physicochemical properties were analyzed in comparison to drug-free multilayer structures. The films were characterized for their physical properties—surface pH, moisture content and moisture absorption capacity, swelling behavior and mucoadhesion. Furthermore, the drug content and the release behavior were investigated.

Acidic or alkaline pH of the dosage form may cause irritation to the buccal mucosa; therefore, surface pH was determined to evaluate the films’ tolerability. Surface pH close to that of the buccal mucosa or the saliva was considered optimum. The surface pH of the films was within the range of salivary pH (6.35–6.80) except for the BZ-loaded sample, which was found to be slightly acidic (5.97 ± 0.15) but still close to the buccal pH 6.4 (Table 2). The drug-free films were neutral; the incorporation of drugs that differ in chemical properties (the acidic TA with pKA 5.11 and the basic BZ and BET with pKa values 9.26 and 9.77 respectively) did not significantly alter the surface pH of the drug-loaded films, suggesting no irritant effect and high biocompatibility of the formulated delivery systems.

The moisture loss of the films was investigated to check their integrity under dry conditions. Usually, moisture loss below 5% is a prerequisite for increased physical stability, but also for a higher moisture absorption capacity, which in turn is associated with improved mucoadhesion. The moisture loss of the samples ranged from 0.84% to 1.95% (Table 2). The least amount of moisture lost during the study was found in TA-loaded samples whereas the drug-free films showed almost 2% moisture loss. Since no hygroscopic excipients (e.g., plasticizers) were used in the formulation, which can retain moisture, it can be assumed that the higher the moisture loss, the higher the initial moisture content of the films. The drug-free samples that are composed only of polymers, retain more moisture during preparation than the drug-loaded samples. Hence, the moisture loss is significantly lower in drug-containing films, probably due to the lower polymer content after drug incorporation. Initial moisture content (moisture loss, respectively) is related to moisture absorption capacity.

Determination of moisture absorption is essential for assessing the physical stability of the films at extremely high humidity, which is characteristic of the oral cavity. There is an inverse relationship between these two parameters: the higher the percentage of moisture loss, the lower the moisture absorption and vice versa. Moisture absorption of the samples was in the range 0.75–4.99% (Table 2) suggesting high physical stability of the samples in a humid environment. These results correlate with the ones obtained in the swelling study.

**Figure 1:**
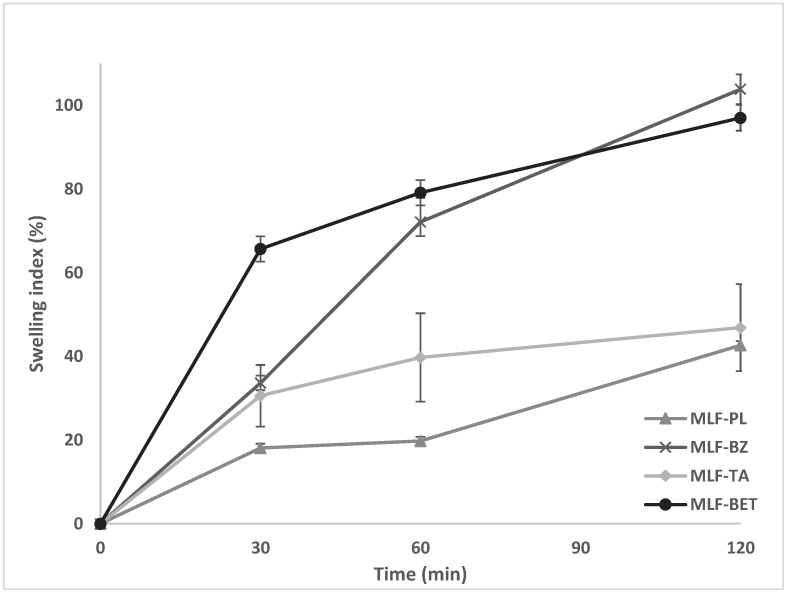
Swelling index of multilayer casein/chitosan films loaded with benzydamine hydrochloride (MLF-BZ), tolfenamic acid (MLF-TA) and betahistine dihydrochloride (MLF-BET) compared to drug-free (placebo) films (MLF-PL), *n* = 3.

The degree of hydration of a polymer structure is an essential property allowing sufficient expansion and entanglement of the polymer chains, thus enhancing interpenetration with mucin, and achieving optimum mucoadhesion. A critical point of hydration of the polymer exists, at which a balance is achieved between optimal swelling and mucoadhesion. The swelling behavior of the multilayer films was investigated gravimetrically in simulated saliva solution (PBS, pH = 6.75). The swelling index of the samples varied greatly, depending on the composition of the films (Figure 1). The lowest values were demonstrated in the drug-free samples (MLF-PL) starting from 18% in the 30th minute of the test and reaching only 42% after the second hour of the study. Incorporation of the model drugs in the polymer films substantially increased the swelling capacity of the samples. The highest swelling index was determined in the films containing BZ and BET (103.85% and 97.01%, respectively). Such values for the swelling index could be considered relevant to buccal drug delivery systems due to the lower risk of excessive polymer erosion yet allowing high drug diffusion rate. The higher swelling index of the drug loaded samples in comparison to the drug-free model could be attributed to the presence of BZ and BET in the finishing chitosan layer. Since these two drugs are freely soluble in aqueous media, they diffuse into the testing fluid (simulated saliva solution) leading to the formation of multiple channels and pores in the chitosan layer. The polymer chains are allowed to hydrate freely, followed by a substantial water uptake. Our hypothesis was confirmed by the swelling profile of MLF-TA. Tolfenamic acid, which is incorporated in the casein layer, being practically insoluble in aqueous media at the experimental pH (6.75), diffuses slowly through the polyelectrolyte network, preventing the formation of voids in the polymer layers for water penetration.

For the development of an effective dosage form for buccal administration, it is of crucial importance to evaluate the mucoadhesive property of the formulation. A formulation that lacks optimal mucoadhesive properties does not adhere to the application site for a sufficient period and is unable to provide the desired therapeutic effect. In the present study, mucoadhesion was analyzed based on the mucin binding capacity of the multilayer films. Due to the presence of positively charged amino groups in the structure, chitosan interacts with negatively charged molecules (e.g., mucin). Such electrostatic interaction is expected to manifest into significant adherence of the delivery system to mucosal tissues. The results from the mucin adsorption study are presented in Table 1. The samples exhibited satisfactory mucoadhesion, adsorbing 60–70% mucin. The smallest amount of mucin (60.26%) was adsorbed by the drug-free multilayer films. Drug loading in the samples lead to a slight increase in mucin adsorption (65.04–70.84%) with comparable values regardless of the properties of the drugs used. It turned out that the BZ-loaded samples showed similar mucoadhesion as TA-loaded ones even though BZ was incorporated in the chitosan layers while TA was included in the subterminal casein layers. In addition, it could be expected that the samples containing BZ and BET would manifest lower mucoadhesion compared to the drug-free films, due to the presence of drug molecules in the final chitosan layer, which minimizes the polymer contact surface for interaction with mucin. These assumptions were refuted by the obtained results and lead to the conclusion that the polymer structure of the multilayer films is the most crucial factor for mucoadhesion. A possible explanation of the results could be associated with the formation of a more loose and porous architecture of the multilayers upon incorporation of drug molecules. Such a structure would allow a large area for mucin penetration and interaction with chitosan.

**Figure 2:**
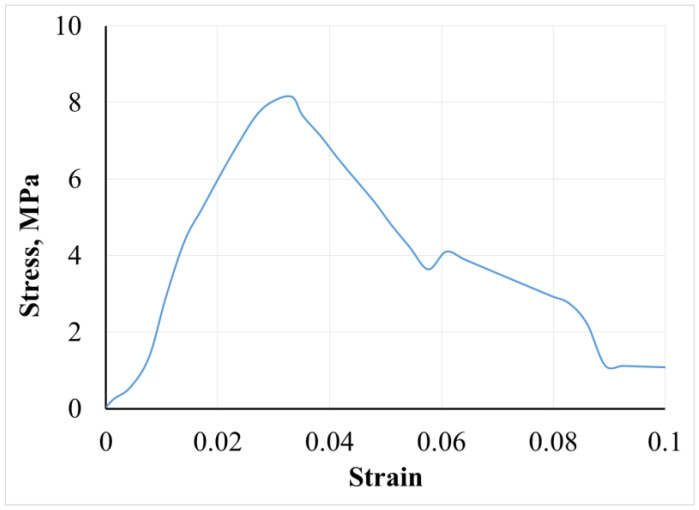
Stress–strain curve of drug-free casein-chitosan polyelectrolyte multilayer film.

The mechanical properties of drug-free multilayer films were studied at uniaxial tensile deformation and the main characteristics (stress at break, strain at break and Young’s modulus) were calculated from the stress–strain curve, presented in Figure 2. The sample is characterized by a normal fracture stress (8.72 ± 1.38) MPa and a relative fracture strain of 0.036 ± 0.001 (3.6 ± 0.1%). The Young’s modulus is determined by linear regression of the stress–strain dependence for strain values less than 3%. Its value is (180 ± 4) MPa, suggesting an optimum mechanical strength of the developed multilayers.

**Figure 3:**
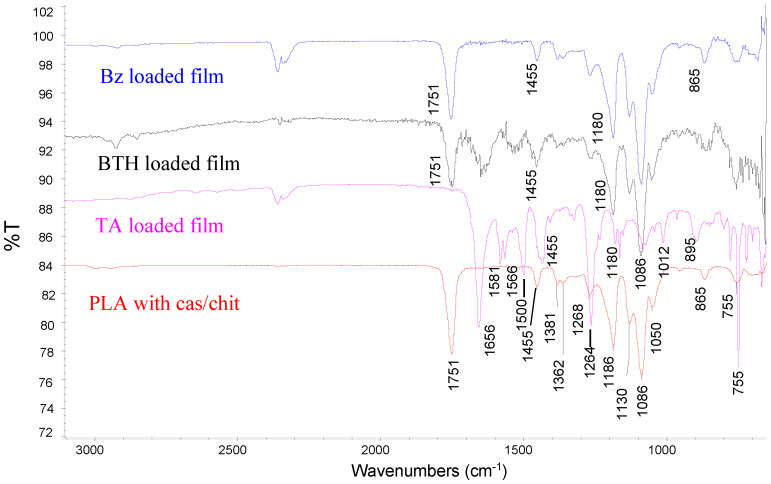
ATR-FTIR spectra of casein-chitosan polyelectrolyte multilayer films.

The FTIR spectra (Figure 3) were acquired by Nicolet iS10 system in the range from 650 to 4000 cm^−1^. In all the spectra the peaks at 1750 cm^−1^ and 1186 cm^−1^ correspond to C=O and C-O stretching of an ester group (PLA). Amide I and amide II bands, characteristic for casein, appear in the region 1680–1500 cm^−1^. The bands at 1130 cm^−1^, 1086 cm^−1^ and 1051 cm^−1^ resulted from the C-O stretching in C-OH and C-O-C bonds in substrate, casein, and glucosamine ring of chitosan. The band at 978 cm^−1^ (dianionic phosphates), that is present in the casein spectrum, is not seen, suggesting an interaction with NH3+ of chitosan. Loading of benzydamin, betahistine and tolfenamic acid is confirmed by increasing the absorption in the region 1640–1450 cm^−1^ and 2000–1800 cm^−1^ due to the aromatic rings of the drugs.

**Figure 4:**
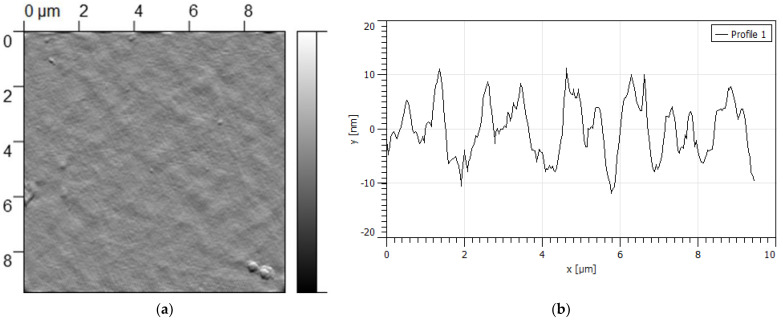
Atomic force microscopy (AFM) image of drug-free multilayer film MLF-PL (**a**), and cross-section (**b**).

AFM was used to investigate the multilayer films’ surface morphology. AFM image of MLF-PL (Figure 4) indicates that the film is characterized with uniform surface morphology and lower surface roughness-*Rq* = (5.125 ± 0.63) nm. The size of the morphological units is about 500 nm in diameter. The densely packed structure for films implies a good entrapment efficiency of drugs, suggesting capacity for prolonged release [29].

**Figure 5:**
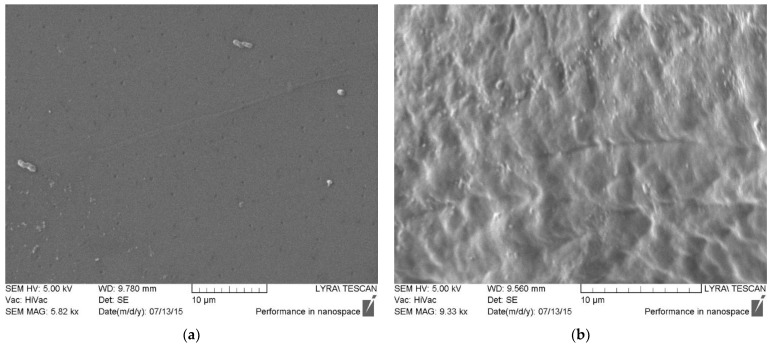
SEM images of PLA substrate (**a**), and drug-free multilayer film MLF-PL (**b**).

Scanning electron microscopy (Figure 5) revealed the presence of numerous morphological units on the surface of the multilayer films, which are due to polyelectrolyte interactions between chitosan and casein and the formation of interpenetrating layers. With polymer deposition on the PLA substrates, long “tails” appear in the structure, which are observed as lamellar formations on the surface.

**Figure 6:**
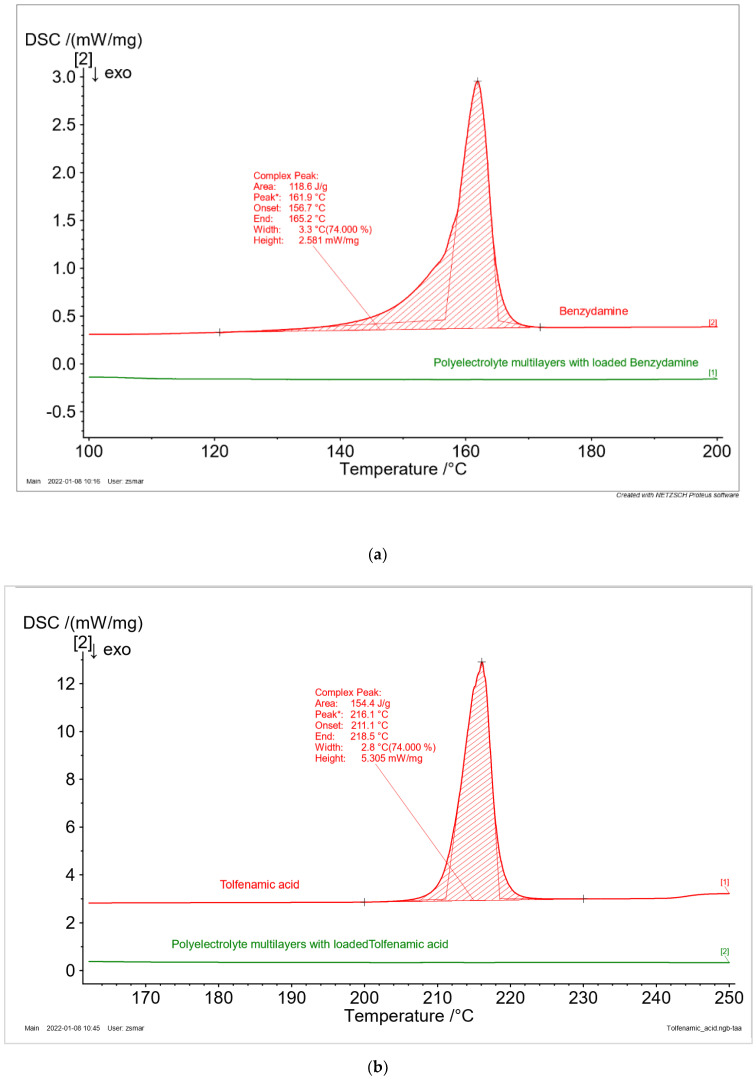

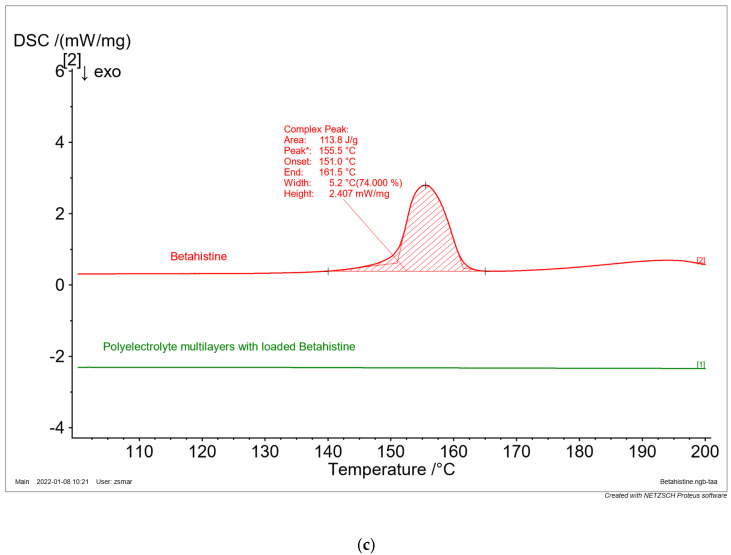
DSC thermogram of multilayer casein/chitosan films loaded with benzydamine hydrochloride (**a**), tolfenamic acid (**b**) and betahistine dihydrochloride (**c**) compared to pure drugs.

The phase state of the drugs (crystal or amorphous) is one of the most important physical parameters, which influence their bioavailability. The thermal behavior of drug-free and BZ, TA, and BET-loaded samples were examined by the method of DSC. The thermograms of the pure drugs (Figure 6) are characterized by a narrow endothermic peak, which could be recognized as a melting phenomenon. Therefore, it is confirmed that all the investigated drugs are crystals in neat form. The values for their melting temperature and enthalpy of fusion are presented in Table 3. No peaks were observed for the drugs loaded in polyelectrolyte multilayers. Hence, it might be assumed that the entrapment of the drugs in the polyelectrolyte matrix change their phase from crystal to amorphous. The received experimental data are in good agreement with the literature [30,31].

One of the most important aspects of this study was to investigate how drugs with different physico-chemical properties would affect the characteristics of polyelectrolyte multilayer films based on casein and chitosan. The results demonstrated substantial variations in drug content (Table 2). It was found that BZ-loaded films of total area 4 cm^2^. contained only 90.48 µg. Identical results were obtained for BET-loaded samples (95.71 µg). This similarity could be explained with the common physical characteristics of the model drugs. Both benzydamine hydrochloride and betahistine dihydrochloride are freely soluble in aqueous media. Given the preparation technique, the loss of the drug could be attributed to their aqueous solubility. Probably, significant amounts of BZ and BET have been washed away during the rinsing step after the polymer deposition. Following the same procedure, TA was incorporated in the casein layers, achieving a ten times higher drug content compared to the other two model drugs (977.33 µg), thus supporting the above hypothesis. Because TA is practically insoluble at a neutral pH, once incorporated with casein, it will not be washed away during rinsing, maintaining a maximum drug load in the multilayers.

**Figure 7:**
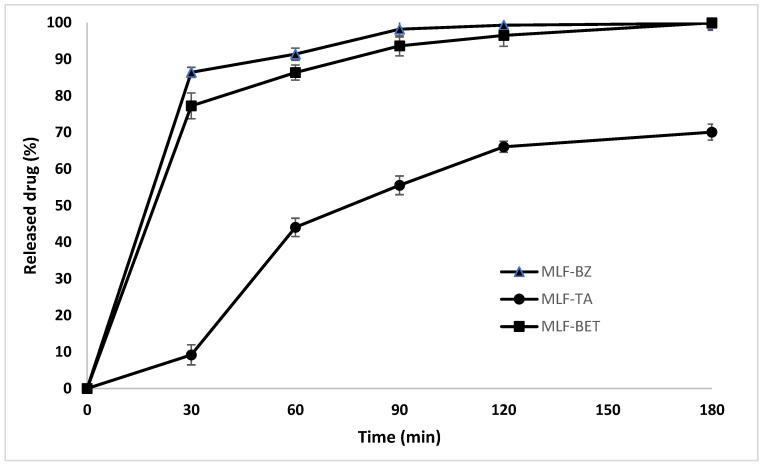
Release profiles of benzydamine hydrochloride (MLF-BZ), tolfenamic acid (MLF-TA) and betahistine dihydrochloride (MLF-BET), *n* = 3.

Typically, polymer structures like the fabricated multilayer films demonstrate prolonged dissolution patterns due to gradual diffusion of the drug molecules through the polymer matrix. However, the results from our study did not confirm this concept (Figure 7). The release patterns of BZ and BET are biphasic, with a clearly defined initial phase characterized by an extensive burst effect within the first 30 min. In the second phase there is a delay, and the release rate is lower. The rapid drug release from MLF-BZ and MLF-BET may be due to faster swelling of the polymer network, resulting in more void space. Indeed, the drug release profiles resemble greatly the swelling study patterns, suggesting the strong dependence of drug release on the swelling behavior of polymer delivery systems. This assumption is supported by the release profile of MLF-TA. In addition to the poor TA solubility, MLF-TA samples are less swellable, and achieve prolonged but incomplete drug release (70.08%).

## 4. Conclusions

Within the scope of this study, multilayer buccal films were prepared using the oppositely charged polyelectrolytes casein and chitosan. The polymers were deposited on corona pre-charged PDLA backing supports by LbL technique and benzydamine, tolfenamic acid and betahistine were incorporated in the multilayers. The samples’ surface pH was within the range of salivary pH suggesting high biotolerability. The moisture content and the moisture absorption studies demonstrated optimum physical stability in an extremely humid and dry environment. Swelling behavior, drug content and drug release were dependent on the physical properties of the loaded drugs. The developed multilayer films appear to be a suitable delivery system mainly for sparingly soluble active pharmaceutical ingredients due to the higher drug loading and the possibility of prolonging the drug release.

## Figures and Tables

**Table 1:** Chromatographic conditions for determination of benzydamine, tolfenamic acid and betahistine. The mobile phase for BZ and BET was a mixture of Phase A and Phase B (80:20). pH was adjusted to 10.5 with triethyl amine.

Model Drug	Mobile Phase		UV Detection	Retention Time (min)
	Phase A	Phase B		
Benzydamine	water	acetonitril	260 nm	4.01 ± 0.10
Betahistine	water	acetonitril	307 nm	4.04 ± 0.10
Tolfenamic acid	60% acetonytril with 0.1% formic acid		280 nm	7.39 ± 0.11

**Table 2:** Characteristics of multilayer casein/chitosan films loaded with benzydamine hydrochloride (MLF-BZ), tolfenamic acid (MLF-TA) and betahistine dihydrochloride (MLF-BET) compared to drug-free (placebo) films (MLF-PL), *n* = 3.

Sample Code	Model Drug	Surface pH ± SD	Moisture Loss % ± SD	Moisture Absorption % ± SD	Mucin Adsorption% ± SD	Drug Content µg ± SD
MLF-PL	-	6.36 ± 0.07	1.95 ± 0.28	0.75 ± 0.09	60.26 ± 0.28	-
MLF-BZ	Benzydamine hydrochloride	5.97 ± 0.15	1.17 ± 0.24	2.46 ± 0.24	65.04 ± 1.12	90.48 ± 2.65
MLF-TA	Tolfenamic acid	6.80 ± 0.16	0.84 ± 0.11	4.99 ± 0.91	66.32 ± 1.02	977.33 ± 11.76
MLF-BET	Betahistine dihydrochloride	6.51 ± 0.13	1.27 ± 0.12	3.04 ± 0.69	70.84 ± 0.36	95.71 ± 3.21

**Table 3:** Differential scanning calorimetry (DSC) parameters (melting temperature and enthalpy of fusion) of the model drugs incorporated in polyelectrolyte multilayer films.

Model Drug	Melting Temperature			Enthalpy of Fusion, J/g
	T_on_, °C	T_p_, °C	T_end_, °C	
Benzydamine	156.7	161.9	165.2	118.6
Tolfenamic acid	211.1	216.1	218.5	154.4
Betahistine	151.0	155.5	161.5	113.8

## Data Availability

Not applicable.
